# 2,2′-(1,4-Phenyl­ene)bis­(propane-2,2-di­yl) bis­(benzodi­thio­ate)

**DOI:** 10.1107/S160053681303465X

**Published:** 2014-01-08

**Authors:** Rodolfo Moreno-Fuquen, Carlos Grande, Rigoberto C. Advincula, Juan C. Tenorio, Javier Ellena

**Affiliations:** a Departamento de Química - Facultad de Ciencias, Universidad del Valle, Apartado 25360, Santiago de Cali, Colombia; b Programa de Ingenieria Agroindustrial, Universidad San Buenaventura, AA 7154, Santiago de Cali, Colombia; c Case Western Reserve University, Department of Macromolecular Science and Engineering, 2100 Adelbert Road, Kent Hale Smith Bldg, Cleveland, Ohio 44106, USA; d Instituto de Física de São Carlos, IFSC, Universidade de São Paulo, USP, São Carlos, SP, Brazil

## Abstract

The title compound, C_26_H_26_S_4_, shows a dihedral angle of 76.64 (15)° between the central and peripheral benzene rings. An inversion center is located at the centroid of the thio­benzoyl ring. In the crystal, weak C—H⋯S inter­actions form *C*(5) chains along [001]. There are no classical hydrogen bonds.

## Related literature   

For control of the behavior of polymerization reactions, see: Patton *et al.* (2005 ▶); You *et al.* (2007 ▶); Pafiti *et al.* (2010 ▶). For radical polymerization with RAFT reactions, see: Le *et al.* (1998 ▶). For telechelic polymers, see: Tasdelen *et al.* (2011 ▶); Goethals (1989 ▶). For hydrogen bonding, see: Nardelli (1995 ▶). For graph-set motifs, see: Etter (1990 ▶). For standard bond lengths, see: Allen *et al.* (1987 ▶).
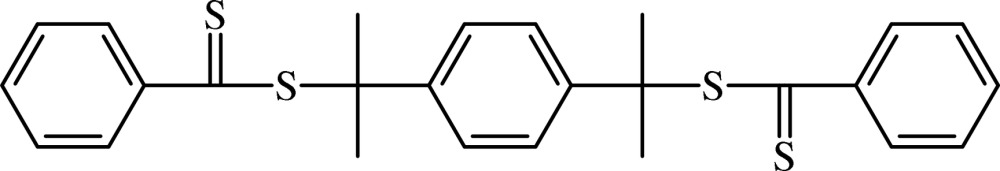


## Experimental   

### 

#### Crystal data  

C_26_H_26_S_4_
*M* *_r_* = 466.75
Monoclinic, 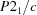

*a* = 8.6981 (6) Å
*b* = 11.7074 (7) Å
*c* = 12.5612 (6) Å
β = 107.626 (4)°
*V* = 1219.08 (13) Å^3^
*Z* = 2
Mo *K*α radiation
μ = 0.40 mm^−1^
*T* = 295 K
0.41 × 0.29 × 0.16 mm

#### Data collection  

Bruker–Nonius KappaCCD diffractometer
Absorption correction: multi-scan (*SADABS*; Sheldrick, 2002 ▶) *T*_min_ = 0.874, *T*_max_ = 0.939
4051 measured reflections
2160 independent reflections
1775 reflections with *I* > 2σ(*I*)
*R*_int_ = 0.021

#### Refinement  

*R*[*F* ^2^ > 2σ(*F* ^2^)] = 0.032
*wR*(*F* ^2^) = 0.091
*S* = 1.03
2160 reflections
136 parameters
H-atom parameters constrained
Δρ_max_ = 0.19 e Å^−3^
Δρ_min_ = −0.22 e Å^−3^

### 

Data collection: *COLLECT* (Hooft, 2004 ▶); cell refinement: *SCALEPACK* (Otwinowski & Minor, 1997 ▶); data reduction: *DENZO* (Otwinowski & Minor, 1997 ▶) and *SCALEPACK*; program(s) used to solve structure: *SHELXS97* (Sheldrick, 2008 ▶); program(s) used to refine structure: *SHELXL97* (Sheldrick, 2008 ▶); molecular graphics: *ORTEP-3 for Windows* (Farrugia, 2012 ▶) and *Mercury* (Macrae *et al.*, 2006 ▶); software used to prepare material for publication: *WinGX* (Farrugia, 2012 ▶).

## Supplementary Material

Crystal structure: contains datablock(s) I, global. DOI: 10.1107/S160053681303465X/kj2235sup1.cif

Structure factors: contains datablock(s) I. DOI: 10.1107/S160053681303465X/kj2235Isup2.hkl

Supporting information file. DOI: 10.1107/S160053681303465X/kj2235Isup3.cml

CCDC reference:

Additional supporting information:  crystallographic information; 3D view; checkCIF report

## Figures and Tables

**Table 1:** Hydrogen-bond geometry (Å, °)

*D*—H⋯*A*	*D*—H	H⋯*A*	*D*⋯*A*	*D*—H⋯*A*
C5—H5⋯S2^i^	0.93	2.94	3.489 (2)	119

Symmetry code: (i) 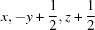.

**Fig. 1.**
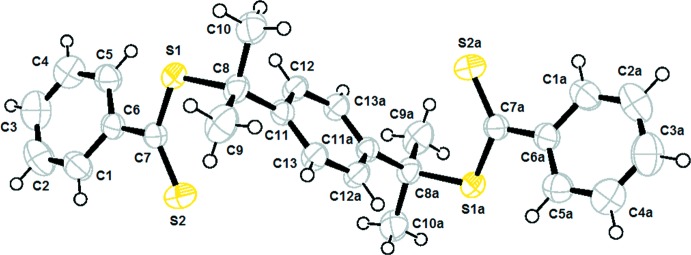
An ORTEP-3 (Farrugia, 2012) plot of the title compound with displacement ellipsoids drawn at the 50% probability level. H atoms are shown as spheres of arbitrary radius. Atoms labelled with suffix "a" are generated by an inversion center via operation (1-x, 1-y, -z).

**Fig. 2.**
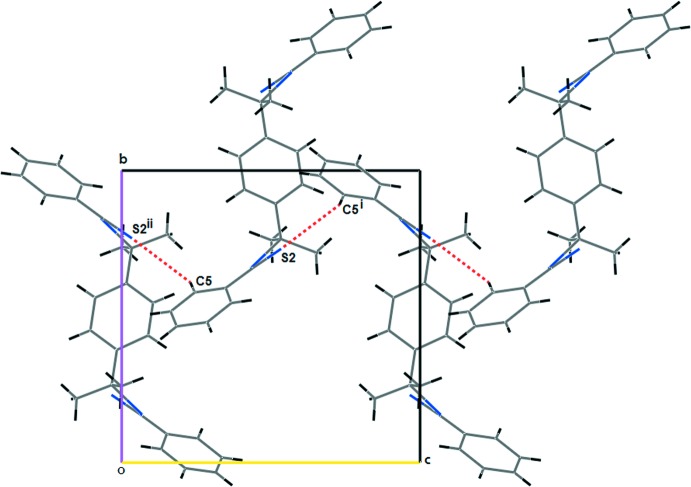
Part of the crystal structure of the title compound, showing the formation of chains of molecules running along [001]. Symmetry code: (i) x,-y + 1/2,z + 1/2. (ii) x,-y + 1/2,z - 1/2.

## supplementary crystallographic information

### 1. Comment

The title compound belongs to a series of difunctional compounds that can be used to control the behavior of polymerization reactions to produce straight forward functional telechelic polymers in one pot (Patton *et al.*, 2005; You *et al.*, 2007; Pafiti *et al.*, 2010). They are also used in radical polymerization with RAFT (reversible addition fragmentation chain transfer) reactions (Le *et al.*, 1998). Telechelic polymers, defined as macromolecules with two reactive end groups, have been used for multiple purposes (Tasdelen *et al.*, 2011) including block copolymer synthesis (Goethals, 1989). A perspective view of the molecule of the title compund, showing the atomic numbering scheme, is given in Fig. 1. Bond lengths and angles in the title compound have normal values (Allen *et al.*, 1987). The molecular system has an inversion center and it is located at the center of the thiobenzoyl ring. The benzene rings bridged by the thio (C6—C7—S1—C8—C11) moiety are tilted to each other by a dihedral angle of 76.64 (15)°. The crystal packing shows no classical hydrogen bonds and it is stabilized by weak C—H···S intermolecular interactions, forming C(5) chains (Etter, 1990) along [001] (see Fig. 2; Etter, 1990). The C5 atom of the benzene ring at (*x*,*y*,*z*) acts as hydrogen-bond donors to S2 atom at (*x*, -*y* + 1/2, *z* - 1/2) (see Table 1; Nardelli, 1995).

### 2. Experimental

The synthesis of the mentioned compound was accomplished following a procedure already reported (Le *et al.*, 1998; Patton *et al.*, 2005). A mixture of dithiobenzoic acid (5.00 g, 32.4 mmol) and 1,4-diisopropenylbenzene (2.44 g, 15.4 mmol) in carbon tetrachloride (40 ml) was heated at 348 K for 20 h. The volatiles were removed under reduced pressure and the oily product was mixed with 1:2 diethyl ether/hexane to isolate the product as a pink solid (40%).

### 3. Refinement

All H-atoms were placed in calculated positions [C—H= 0.95 Å for aromatic and C—H= 0.96 Å for methyl group] and refined with *U*_iso_(H) 1.2 and 1.5 times *U*_eq_ of the parent atom, respectively.

### Crystal data

**Table:**

C_26_H_26_S_4_	*Z* = 2
*M**_r_* = 466.75	*F*(000) = 492
Monoclinic, *P*2_1_/*c*	*D*_x_ = 1.272 Mg m^−^^3^
Hall symbol: -P 2ybc	Mo *K*α radiation, λ = 0.71073 Å
*a* = 8.6981 (6) Å	Cell parameters from 4051 reflections
*b* = 11.7074 (7) Å	µ = 0.40 mm^−^^1^
*c* = 12.5612 (6) Å	*T* = 295 K
β = 107.626 (4)°	Block, pink
*V* = 1219.08 (13) Å^3^	0.41 × 0.29 × 0.16 mm

### Data collection

**Table:**

Bruker–Nonius KappaCCD diffractometer	2160 independent reflections
Radiation source: fine-focus sealed tube	1775 reflections with *I* > 2σ(*I*)
Graphite monochromator	*R*_int_ = 0.021
CCD rotation images, thick slices scans	θ_max_ = 25.1°, θ_min_ = 3.0°
Absorption correction: multi-scan (*SADABS*; Sheldrick, 2002)	*h* = −10→10
*T*_min_ = 0.874, *T*_max_ = 0.939	*k* = −12→13
4051 measured reflections	*l* = −14→14

### Refinement

**Table:**

Refinement on *F*^2^	Primary atom site location: structure-invariant direct methods
Least-squares matrix: full	Secondary atom site location: difference Fourier map
*R*[*F*^2^ > 2σ(*F*^2^)] = 0.032	Hydrogen site location: inferred from neighbouring sites
*wR*(*F*^2^) = 0.091	H-atom parameters constrained
*S* = 1.03	*w* = 1/[σ^2^(*F*_o_^2^) + (0.0431*P*)^2^ + 0.3633*P*] where *P* = (*F*_o_^2^ + 2*F*_c_^2^)/3
2160 reflections	(Δ/σ)_max_ < 0.001
136 parameters	Δρ_max_ = 0.19 e Å^−^^3^
0 restraints	Δρ_min_ = −0.22 e Å^−^^3^

### Special details

**Table:**

Geometry. All e.s.d.'s (except the e.s.d. in the dihedral angle between two l.s. planes) are estimated using the full covariance matrix. The cell e.s.d.'s are taken into account individually in the estimation of e.s.d.'s in distances, angles and torsion angles; correlations between e.s.d.'s in cell parameters are only used when they are defined by crystal symmetry. An approximate (isotropic) treatment of cell e.s.d.'s is used for estimating e.s.d.'s involving l.s. planes.
Refinement. Refinement of *F*^2^ against ALL reflections. The weighted *R*-factor *wR* and goodness of fit *S* are based on *F*^2^, conventional *R*-factors *R* are based on *F*, with *F* set to zero for negative *F*^2^. The threshold expression of *F*^2^ > σ(*F*^2^) is used only for calculating *R*-factors(gt) *etc*. and is not relevant to the choice of reflections for refinement. *R*-factors based on *F*^2^ are statistically about twice as large as those based on *F*, and *R*- factors based on ALL data will be even larger.

### Fractional atomic coordinates and isotropic or equivalent isotropic displacement parameters (Å^2^)

**Table:**

	*x*	*y*	*z*	*U*_iso_*/*U*_eq_	
S1	0.50091 (6)	0.16376 (4)	0.06933 (4)	0.04958 (17)	
S2	0.75578 (8)	0.23202 (6)	−0.03441 (5)	0.0685 (2)	
C1	0.9423 (3)	0.0464 (2)	0.13247 (19)	0.0610 (6)	
H1	0.9617	0.0565	0.0642	0.073*	
C2	1.0454 (3)	−0.0191 (2)	0.2131 (2)	0.0754 (7)	
H2	1.1332	−0.0541	0.1990	0.090*	
C3	1.0198 (3)	−0.0335 (2)	0.3155 (2)	0.0754 (7)	
H3	1.0904	−0.0778	0.3704	0.091*	
C4	0.8906 (3)	0.0175 (2)	0.33591 (18)	0.0644 (6)	
H4	0.8736	0.0081	0.4050	0.077*	
C5	0.7852 (3)	0.08278 (17)	0.25504 (16)	0.0526 (5)	
H5	0.6975	0.1171	0.2699	0.063*	
C6	0.8086 (2)	0.09805 (15)	0.15112 (16)	0.0456 (4)	
C7	0.6960 (2)	0.16592 (15)	0.06088 (16)	0.0462 (5)	
C8	0.3740 (2)	0.26543 (15)	−0.03377 (16)	0.0462 (4)	
C9	0.3421 (3)	0.21941 (19)	−0.15129 (18)	0.0668 (6)	
H9A	0.2986	0.1436	−0.1554	0.100*	
H9B	0.4413	0.2174	−0.1699	0.100*	
H9C	0.2663	0.2681	−0.2030	0.100*	
C10	0.2164 (3)	0.26165 (19)	−0.0017 (2)	0.0628 (6)	
H10A	0.2361	0.2906	0.0727	0.094*	
H10B	0.1789	0.1842	−0.0050	0.094*	
H10C	0.1360	0.3078	−0.0529	0.094*	
C11	0.4441 (2)	0.38607 (15)	−0.01603 (14)	0.0388 (4)	
C12	0.4914 (2)	0.43533 (16)	0.08868 (15)	0.0446 (4)	
H12	0.4861	0.3926	0.1499	0.054*	
C13	0.4535 (2)	0.45299 (16)	−0.10490 (15)	0.0439 (4)	
H13	0.4222	0.4225	−0.1766	0.053*	

### Atomic displacement parameters (Å^2^)

**Table:**

	*U*^11^	*U*^22^	*U*^33^	*U*^12^	*U*^13^	*U*^23^
S1	0.0527 (3)	0.0368 (3)	0.0662 (3)	0.0003 (2)	0.0284 (2)	0.0081 (2)
S2	0.0734 (4)	0.0679 (4)	0.0797 (4)	0.0051 (3)	0.0461 (3)	0.0187 (3)
C1	0.0580 (13)	0.0617 (13)	0.0683 (13)	0.0053 (11)	0.0264 (11)	−0.0067 (11)
C2	0.0560 (14)	0.0766 (17)	0.0882 (18)	0.0152 (12)	0.0138 (13)	−0.0131 (14)
C3	0.0697 (16)	0.0678 (16)	0.0714 (16)	0.0058 (13)	−0.0046 (13)	−0.0023 (13)
C4	0.0687 (15)	0.0667 (14)	0.0507 (12)	−0.0063 (12)	0.0077 (11)	−0.0045 (11)
C5	0.0566 (12)	0.0493 (11)	0.0532 (11)	−0.0037 (9)	0.0186 (10)	−0.0082 (9)
C6	0.0502 (10)	0.0342 (9)	0.0551 (11)	−0.0045 (8)	0.0200 (9)	−0.0074 (8)
C7	0.0551 (11)	0.0340 (9)	0.0564 (11)	−0.0011 (8)	0.0270 (9)	−0.0042 (8)
C8	0.0493 (11)	0.0349 (9)	0.0540 (11)	−0.0059 (8)	0.0150 (9)	−0.0007 (8)
C9	0.0871 (17)	0.0466 (12)	0.0601 (13)	−0.0201 (12)	0.0124 (12)	−0.0129 (10)
C10	0.0485 (12)	0.0508 (12)	0.0889 (16)	−0.0078 (9)	0.0207 (12)	0.0033 (11)
C11	0.0379 (9)	0.0333 (9)	0.0461 (10)	−0.0006 (7)	0.0140 (8)	−0.0014 (8)
C12	0.0551 (11)	0.0391 (10)	0.0414 (10)	−0.0035 (9)	0.0171 (8)	0.0040 (8)
C13	0.0524 (11)	0.0396 (10)	0.0391 (9)	−0.0037 (8)	0.0129 (8)	−0.0040 (8)

### Geometric parameters (Å, º)

**Table:**

S1—C7	1.7325 (19)	C8—C9	1.516 (3)
S1—C8	1.857 (2)	C8—C11	1.528 (2)
S2—C7	1.6366 (19)	C8—C10	1.541 (3)
C1—C2	1.367 (3)	C9—H9A	0.9600
C1—C6	1.393 (3)	C9—H9B	0.9600
C1—H1	0.9300	C9—H9C	0.9600
C2—C3	1.380 (4)	C10—H10A	0.9600
C2—H2	0.9300	C10—H10B	0.9600
C3—C4	1.364 (3)	C10—H10C	0.9600
C3—H3	0.9300	C11—C12	1.380 (2)
C4—C5	1.376 (3)	C11—C13	1.386 (2)
C4—H4	0.9300	C12—C13^i^	1.386 (3)
C5—C6	1.392 (3)	C12—H12	0.9300
C5—H5	0.9300	C13—C12^i^	1.386 (3)
C6—C7	1.484 (3)	C13—H13	0.9300
			
C7—S1—C8	109.55 (9)	C9—C8—S1	110.25 (14)
C2—C1—C6	120.9 (2)	C11—C8—S1	111.34 (13)
C2—C1—H1	119.6	C10—C8—S1	100.81 (13)
C6—C1—H1	119.6	C8—C9—H9A	109.5
C1—C2—C3	120.3 (2)	C8—C9—H9B	109.5
C1—C2—H2	119.9	H9A—C9—H9B	109.5
C3—C2—H2	119.9	C8—C9—H9C	109.5
C4—C3—C2	119.8 (2)	H9A—C9—H9C	109.5
C4—C3—H3	120.1	H9B—C9—H9C	109.5
C2—C3—H3	120.1	C8—C10—H10A	109.5
C3—C4—C5	120.5 (2)	C8—C10—H10B	109.5
C3—C4—H4	119.8	H10A—C10—H10B	109.5
C5—C4—H4	119.8	C8—C10—H10C	109.5
C4—C5—C6	120.7 (2)	H10A—C10—H10C	109.5
C4—C5—H5	119.7	H10B—C10—H10C	109.5
C6—C5—H5	119.7	C12—C11—C13	117.27 (16)
C5—C6—C1	117.94 (19)	C12—C11—C8	121.06 (16)
C5—C6—C7	122.36 (17)	C13—C11—C8	121.55 (16)
C1—C6—C7	119.70 (18)	C11—C12—C13^i^	121.62 (16)
C6—C7—S2	121.99 (14)	C11—C12—H12	119.2
C6—C7—S1	112.15 (13)	C13^i^—C12—H12	119.2
S2—C7—S1	125.85 (13)	C11—C13—C12^i^	121.12 (17)
C9—C8—C11	114.68 (16)	C11—C13—H13	119.4
C9—C8—C10	109.22 (18)	C12^i^—C13—H13	119.4
C11—C8—C10	109.63 (16)		
			
C6—C1—C2—C3	−1.1 (4)	C7—S1—C8—C9	71.76 (16)
C1—C2—C3—C4	0.3 (4)	C7—S1—C8—C11	−56.71 (15)
C2—C3—C4—C5	0.3 (4)	C7—S1—C8—C10	−172.92 (13)
C3—C4—C5—C6	−0.1 (3)	C9—C8—C11—C12	−174.69 (18)
C4—C5—C6—C1	−0.7 (3)	C10—C8—C11—C12	62.0 (2)
C4—C5—C6—C7	178.79 (18)	S1—C8—C11—C12	−48.6 (2)
C2—C1—C6—C5	1.2 (3)	C9—C8—C11—C13	9.4 (3)
C2—C1—C6—C7	−178.2 (2)	C10—C8—C11—C13	−113.8 (2)
C5—C6—C7—S2	151.88 (16)	S1—C8—C11—C13	135.50 (16)
C1—C6—C7—S2	−28.7 (3)	C13—C11—C12—C13^i^	−0.2 (3)
C5—C6—C7—S1	−28.9 (2)	C8—C11—C12—C13^i^	−176.23 (17)
C1—C6—C7—S1	150.59 (16)	C12—C11—C13—C12^i^	0.2 (3)
C8—S1—C7—C6	172.46 (12)	C8—C11—C13—C12^i^	176.21 (17)
C8—S1—C7—S2	−8.33 (16)		

Symmetry code: (i) −*x*+1, −*y*+1, −*z*.

### Hydrogen-bond geometry (Å, º)

**Table:**

*D*—H···*A*	*D*—H	H···*A*	*D*···*A*	*D*—H···*A*
C5—H5···S2^ii^	0.93	2.94	3.489 (2)	119

Symmetry code: (ii) *x*, −*y*+1/2, *z*+1/2.
